# Fabrication and Performance of a Ta_2_O_5_ Thin Film pH Sensor Manufactured Using MEMS Processes

**DOI:** 10.3390/s23136061

**Published:** 2023-06-30

**Authors:** Yuzhen Guo, Zengxing Zhang, Bin Yao, Jin Chai, Shiqiang Zhang, Jianwei Liu, Zhou Zhao, Chenyang Xue

**Affiliations:** 1 State Key Laboratory of Dynamic Measurement Technology, North University of China, Taiyuan 030051, China; s202206063@st.nuc.edu.cn (Y.G.); s202106040@st.nuc.edu.cn (B.Y.); sz202206092@st.nuc.edu.cn (S.Z.); s202206098@st.nuc.edu.cn (J.L.); b200621@st.nuc.edu.cn (Z.Z.); 2 Pen-Tung Sah Institute of Micro-Nano Science and Technology, Xiamen University, Xiamen 361005, China; 3 Tan Kah Kee Innovation Laboratory, Xiamen 361005, China; 4 Xiamen Zehuo Industry Digital Research Institute Co., Ltd., Xiamen 361005, China; cj@zehuo.cc

**Keywords:** Ta_2_O_5_, capacitive pH sensor, potentiometric method, miniaturization

## Abstract

In this work, a capacitive pH sensor consisting of Ta_2_O_5_ functional film is designed and fabricated by employing MEMS-based procedures. The Ta_2_O_5_ thin film has an amorphous microstructure, and its surface roughness is less than 3.17 nm. A signal processing circuit and a software filtering algorithm are also designed to measure the pH value, thus improving the detection accuracy and anti-interference ability. Good linearity (R^2^ = 0.99904) and sensitivity (63.12 mV/pH) are recorded for the proposed sensing element in the range of pH 2~12. In addition, the sensor’s drift and hysteresis are equal to 5.1 mV and 5.8 mV, respectively. The enhanced sensing performance in combination with the facile miniaturization process, low fabrication cost, and suitability for mass production render the fabricated sensor attractive for applications where pH change measurements in a water environment are required.

## 1. Introduction

Active hydrogen ion concentration can be obtained directly from the pH measurements of a solution [1]. The pH value is essential in the marine ecological physicochemical environment, and is regarded as one of the most basic observation parameters in marine environmental surveys and aquaculture [2]. The continuous dissolution of CO_2_ in the atmosphere causes the concentration of hydrogen ions ($H^{+}$) in the ocean to eventually increase and the concentration of carbonate (${\mathrm{CO}}_{3}^{2-}$) in the seawater to decrease. Consequently, the pH of seawater ($\mathrm{pH}=-\mathrm{lg}\;[H^{+}]$) decreases [3,4,5]. This process is called “ocean acidification”. The absorption of atmospheric carbon dioxide by the marine system dramatically changes the chemical balance of the marine carbonate system, and significantly impacts both marine life and ecosystems [4,6]. Therefore, it is crucial to develop a novel element sensor that can monitor pH changes based on this background.

The fabrication of electrochemical pH sensors has been reported by several authors in recent years [7,8]. A pH sensor based on glass-sensitive electrodes consists of a glass bubble filled with a strong electrolyte and an internal silver chloride electrode, and is often used for accurate pH measurements [9]. However, glass electrodes have several shortcomings such as fragility, high impedance, slow response time and difficult miniaturization. Therefore, they are not suitable in special situations where high temperature, high pressure, high acidity and high alkalinity are present [8]. To address the emerging challenges, the replacement of glass electrodes by non-glass electrodes consisting of sensitive thin film material for the fabrication of electrochemical pH sensors is emerging as a promising solution [2,10]. The development of such types of electrodes is also of great importance for water environmental monitoring. Compared with other types of sensitive layers, metal oxides have the comparative advantages of fast response, long service life, stable mechanical and chemical properties, and simple miniaturization processes [11,12,13]. Therefore, they can be used to detect hydrogen ions and have proven to be the most promising candidate for determining the pH of a solution.

We emphasize that the selection of appropriate materials and substrates, in conjunction with the processing cost and miniaturization aspects, are considered essential issues for the sensor design. The main disadvantage of most current pH sensors is the slow reaction in neutral and alkaline solutions compared to acidic solutions. The main range of pH sensors in practical applications is between 5 and 10, so this problem needs to be overcome. The Nernst response of metal-oxide-based pH sensors depends on the material composition type. Several challenges should be addressed before the large-scale utilization of metal-oxide-based sensors, including material composition, cost, toxicity, integration of the working and reference electrodes on a single substrate, thin film fabrication process, etc. Various metal oxide configurations have been selected as pH-sensitive materials [14,15], including iridium oxide (IrO_2_), aluminum oxide (A1_2_O_3_) and tantalum oxide (Ta_2_O_5_). It is mentioned in [16,17] that Ta_2_O_5_-based sensors have the best sensing characteristics, high sensitivity, good linearity, and good thermal and chemical stability.

Ta_2_O_5_ is used as a sensitive film for pH sensing applications, while its surface uniformity is regarded as the primary factor in improving the performance of pH sensors. Several methods were implemented in the literature for fabricating Ta_2_O_5_ thin films, including screen printing, magnetron sputtering, chemical vapor deposition, etc. [18,19]. Of these methods, the growth of Ta_2_O_5_ thin films through reactive magnetron sputtering technique yields the best sensitivity, response time and miniaturization effect [20,21,22]. Thus, a reactive magnetron sputtering process is used to prepare Ta_2_O_5_ thin films in this work. Moreover, Ta_2_O_5_ thin films are currently used to fabricate pH sensors of ion-sensitive field-effect transistor (ISFET) type [23,24,25] and electrolyte-ion-sensitive membrane-oxide-semiconductor (EIOS) type [10,26]. Due to the film formation on the ISFET-based pH sensor surface, there are still significant outstanding problems such as temperature drift, response hysteresis, considerable noise, poor long-term stability and short service life. On the contrary, the ion-sensitive membrane of the EIOS structure exhibits good stability, high reliability and accessible packaging technology.

Under this direction, an EIOS-type pH sensor is fabricated in this work based on the incorporation of a metal oxide Ta_2_O_5_ functional film. The reactive magnetron sputtering process is also used to deposit Ta_2_O_5_ films. The film’s microstructure is characterized after enforcing an annealing step at 400 °C, where good uniformity is achieved in combination with an amorphous structure. The processing circuit is designed according to the potential method to output the input signal as a voltage value. Alongside that, a recursive average filtering algorithm is designed to further improve accuracy. The sensor’s performance is fully characterized by building a measurement platform. Good linearity (R^2^ = 0.99904) and sensitivity (63.12 mV/pH) values are recorded in the range of pH 2~12. The drift and hysteresis of the sensor are 5.1 mV and 5.8 mV, respectively. The proposed sensing configuration can be mass-produced and miniaturized.

## 2. Working Principle of Sensors

**Figure 1:**
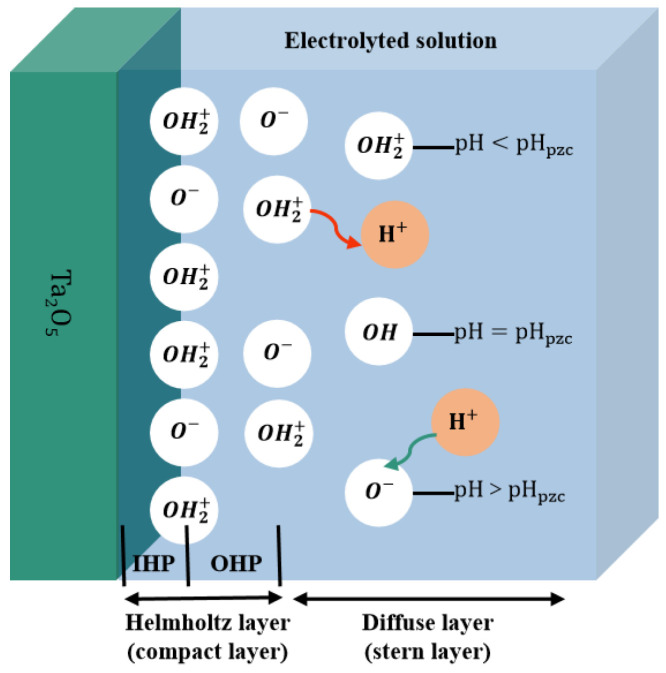
Sensing mechanism of the electrolyte interface.

By considering the measurement solution and interfacial electrochemistry of the EIOS-based sensor, it was confirmed by William et al. [27] that the interaction between the electrolyte and the metal oxide changed the interface potential. As far as the ion exchange process at the interface is concerned, the site-binding theory proposed by Yates et al. [28] is well-established. Figure 1 shows the assumption of the site binding model that when Ta_2_O_5_ is exposed to the electrolyte, its surface is hydrolyzed to form tantalum–hydroxy groups (Ta–OH) [2,29]. The surface is charged by receiving or releasing protons during the reaction with a solution containing $H^{+}/{\mathrm{OH}}^{-}$, which in turn form the $O^{-}$, ${\mathrm{OH}}^{}$ and ${\mathrm{OH}}_{2}^{+}$ surface groups. Figure 1 shows that the charged surface groups at the solution interface create an EDL structure. The EDL structure consists of a Helmholtz layer (compact layer) and a diffusion layer (Stern layer). The Helmholtz layer contains the inner Helmholtz plane (IHP) and the outer Helmholtz plane (OHP).

If the solution is alkaline, the $H^{+}$ will diffuse out of the layer and a negative charge will be generated on the outer side of the membrane. The reverse procedure will occur if the solution is acidic. More specifically, $H^{+}$ will diffuse into the layer and a positive charge will build up on the outer side of the membrane [30]. When the residual charge on the electrode’s surface is zero, the electrode potential is called a zero-charge potential and the pH value that corresponds to the zero-potential point is expressed as pH_pzc_ [31]. The electric double layer effect is formed to maintain the electrical neutrality of the whole system. The reaction process of the Ta_2_O_5_ surface groups is represented by Equations (1) and (2), as follows:

(1) $$\mathrm{Ta}-\mathrm{OH}↔{\mathrm{TaO}}^{-}+H^{+}$$

(2) $${\mathrm{TaOH}}_{2}^{+}↔{\mathrm{Ta}-\mathrm{OH}}^{}+H^{+}$$

A linear relationship exists between the interface potential and the measured substance’s ionic activity under equilibrium conditions, known as the Nernst relationship [2]. The current passing through the sensitive membrane of the potentiometric sensor is zero because the potential change between the working and reference electrodes is directly proportional to the activity of a particular chemical substance in the electrolyte [32]. The reference electrode does not react with the $H^{+}$ concentration in the sample solution. Therefore, the same constant potential will always be produced, against which the pH sensor potential will be measured. The number of hydrogen ions in the solution can be extracted from the potential value between the two electrodes, which provides the solution’s pH value. This potential is a linear function of the hydrogen concentration in the solution, which allows for the execution of quantitative measurements. The relationship between the amount of acid in the solution and the output potential of a pH electrode is provided by the following equation:

(3) $$E=E_{0}-(2.303\frac{\mathrm{RT}}{\mathrm{nF}})\mathrm{pH}$$

where *E* is the equilibrium electrode potential, *E*_0_ represents the standard potential, *R* = 8.314 J/K/mol is the universal gas constant, *T* stands for the absolute thermodynamic temperature, *n* is the number of electrons participating in the electrode reaction and *F* = 9.649 × 10^4^ C/mol denotes the Faraday constant. A quantitative relationship between the electromotive force of the measuring cell and the electrolyte concentration can be observed from Equation (3). The one-electron reaction (*n* = 1) at 25 °C is equal to 59.2 mV for the Nernst factor of 2.303 *RT*/*nF* [12]. A pH sensor with a Ta_2_O_5_ sensing electrode membrane potential exhibits a sub-Nernst response.

## 3. Fabrication and Measurement

### 3.1. Fabrication Process and Package Design

**Figure 2:**
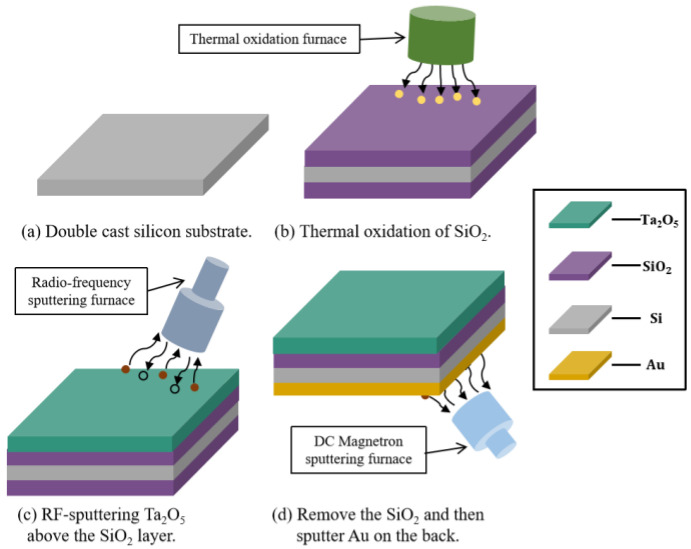
The fabrication process of the pH sensor.

A 525 μm 4-inch P-type <100> silicon wafer is used as the starting material. The sensor is fabricated according to the scheme depicted in Figure 2. First, the silicon wafer is put into acetone and isopropanol for 15 min to remove the natural oxides on the wafer’s surface. Subsequently, 300 nm silicon dioxide (SiO_2_) is thermally grown by dry oxidation at 1100 °C for a duration of three hours. The Ta_2_O_5_ film is deposited by the reactive magnetron sputtering technique, at a 250 W RF power for three hours under the atmosphere of the Ar/O_2_ mixed working gas, with a volume ratio of 4:1 and a base substrate temperature of 350 °C. A 99.999% high-purity Ta_2_O_5_ ceramic material is used as a sputtering target. We underline that the quality of the deposited Ta_2_O_5_ film directly determines the sensor’s performance. The surface morphology and deposit thickness of the Ta_2_O_5_ film are characterized by obtaining scanning electron microscopy (SEM) measurements. Subsequently, the SiO_2_ on the back is etched with a standard BOE solution and a 1:20 diluted BOE solution. Next, 10 nm Ti is deposited via magnetron sputtering as an adhesion layer to improve the adhesion between the Au conductive layer and the Si/SiO_2_ substrate, while a 200 nm gold conductive layer is deposited using the same method as the back contact of the sensor. The electrodes are reserved for the subsequent test leads. Finally, the sample is annealed at 400 °C for two hours, and the crystal structure of the sensor is characterized by employing an X-ray diffractometer (XRD) to ensure the structure of the prepared Ta_2_O_5_ film.

**Figure 3:**
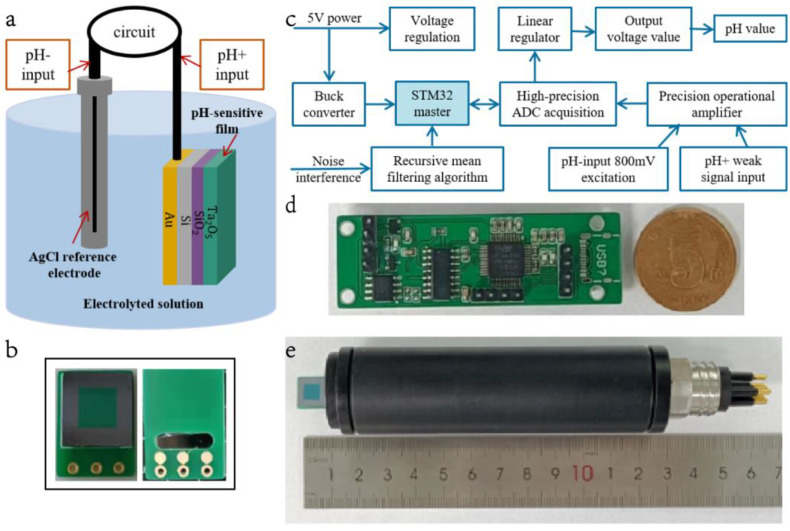
(**a**) pH sensor test principle; (**b**) Structural schematic of the pH sensor; (**c**) Hardware circuit flow chart; (**d**) PCB circuit board; (**e**) Packaged pH sensor used for testing.

When the 4-inch sensor is encapsulated, it is split into a single small sensor of 10 mm × 10 mm. A spot welder is used to draw wires from the conductive gold layer of the electrode to the printed circuit board (PCB). The structural schematic of the sensor is shown in Figure 3b. Except for the Ta_2_O_5_ sensitive film that is directly in contact with the electrolyte, the remaining materials are covered with an ultraviolet-curable adhesive.

### 3.2. Measurement

**Figure 4:**
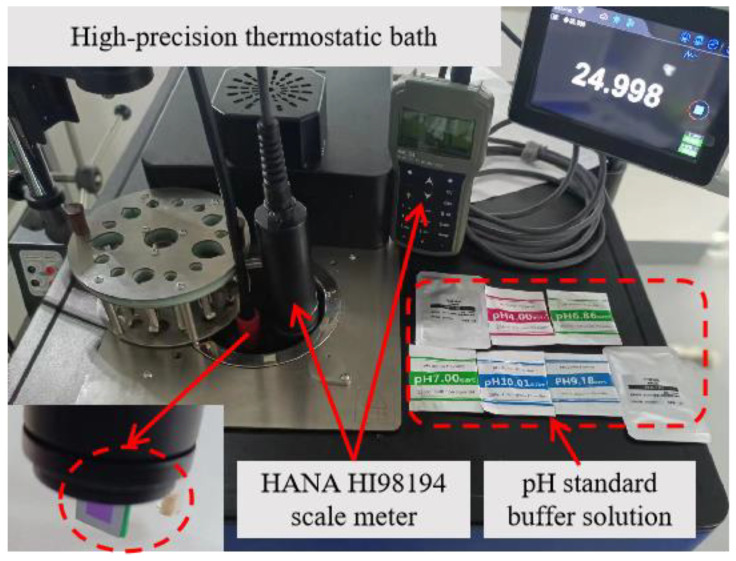
Measurement platform.

The test principle of the sensor is shown in Figure 3a. It can be observed that the reference electrode Ag/AgCl and the pH half-cell must be in the same solution to ensure the correctness of the measurement. The potential of this reference system is defined by the reference electrolyte and the Ag/AgCl reference element. The reference electrolyte has a high ionic concentration, which results in a low electrical resistance [33]. The temperature change also influences the value of the interface potential, and the pH decreases with increasing temperature. For this reason, a high-precision constant temperature bath is used to enforce a stable temperature value at 25 °C for testing. A measurement platform shown in Figure 4 is built based on the aforementioned test requirements, including the pH standard buffer, high-precision thermostatic bath, benchmark pH meter, etc.

The pH electrode is considered an electrochemical sensor, and the pH value results from the measurement of the voltage signal. Figure 3e shows the complete pH sensor. This work uses a two-electrode configuration (signal-electrode-Ta_2_O_5_ and the reference saturated Ag/AgCl electrode) to accurately measure the sensor’s performance in a back-end differential amplifier circuit. Figure 3c,d shows the overall circuit flow chart and the circuit diagram, respectively. A weak voltage signal is generated due to the large input impedance and a small output impedance of the pH electrode. Hence, a differential amplifier circuit must be added after the pH^+^ input of the signal electrode. A single power amplifier chip TLC4502 with a high input impedance is used. The reference electrode pH^−^ provides an excitation signal of 800 mV to pH^+^. Finally, the measured parameter is an analog voltage signal detected through the measuring circuit, while the 16-bit high-precision analog of the digital converter converts the analog value into a digital value that is fed to the STM32 microprocessor. The pH value and voltage of the solution are calculated through the serial output of the DAP simulator. The signal from the pH acquisition circuit can also be stored in the T-Flash Card for a long time through the data acquisition circuit.

The pH electrode signal can be easily affected by an external environmental interference, and the common frequency noise of 50 Hz often appears. Since temperature affects the pH measurement results, we add a temperature compensation algorithm to the hardware program. The performance of the sensor is tested at a constant temperature of 25 °C. When the temperature is different, the slope of pH is also different, so the temperature compensation of the pH usually compensates the slope of the electrode, and automatically compensates the slope of the electrode to the slope under the current test temperature, so as to obtain a pH value under the current test temperature. The design of a filtering algorithm of the program’s recursive average value is regarded as an essential method. The processing idea was to set up a queue of length N, insert the collected data at the end of the queue, and discard the data at the head of the queue at the same time. To further ensure that the N data in the queue is the latest data, the arithmetic means of the N data in the queue are further calculated and used as a valid value. Additionally, a significant change in data transmission can be reflected in the practical value in real-time in a specific acquisition. The proposed system has high smoothness, good real-time performance, and an excellent inhibitory effect on the periodic interference, thus improving the pH value detection accuracy and anti-interference ability.

## 4. Results and Discussion

### 4.1. Microstructural Analysis

**Figure 5:**
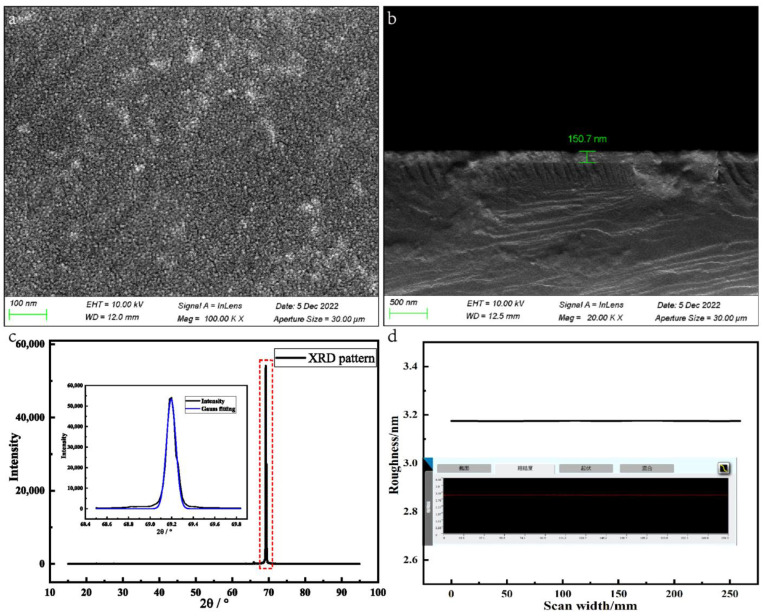
(**a**) SEM surface images of Ta_2_O_5_ film; (**b**) SEM cross-sectional thickness characterization of Ta_2_O_5_ layer: 150.7 nm; (**c**) XRD pattern analysis of the Ta element; (**d**) The average roughness of the Ta_2_O_5_ thin film.

The chip surface and cross-section are examined with a scanning electron microscope (SEM), of which the product model is ZEISS SUPRA-55. Figure 5a shows the surface morphology of Ta_2_O_5_ film in a 100-level ultra-clean environment. Regarding the thin film sputtered at a substrate temperature of 300 °C, no particle gaps are found after observation under a maximum magnification by a factor of 100,000. Figure 5b shows the SEM cross-section, where it can be observed that the thickness of the Ta_2_O_5_ film is 150.7 nm, which meets the fabrication requirements.

X-ray diffraction (XRD) measurements are also obtained for the Ta_2_O_5_ thin film annealed at 400 °C [34]. Compared with the powder diffraction file (PDF), it is found that the peak at 69° can be assigned to the characteristic peak of the metallic Ta. Furthermore, no diffraction peaks related to the existence of any type of Ta_2_O_5_ crystal structure are recorded, which is amorphous, as can be observed from the spectrum displayed in Figure 5c. The transition temperature of the Ta_2_O_5_ film from amorphous to the crystalline state is higher than 400 °C similar to the XRD pattern tested by Ren et al. [18], which is in direct agreement with the measured data.

The manifestation of a smooth and uniform sensor surface is also highly critical for capacitive sensors. A laser confocal microscope (LEXTOLS4100) was used to measure the surface roughness of MEMS-processed samples and observe the surface of Ta_2_O_5_ film. As Figure 5d shows, the average roughness of the Ta_2_O_5_ thin film is less than 3.17 nm, which is mainly attributed to the existence of dispersed nanoparticles. Consequently, it can be argued that the film’s surface is very uniform and smooth, which improves the response time and sensitivity of the sensor.

### 4.2. Testing

Standard buffer solutions with pH values of 1.68, 4.00, 6.86, 7.00, 9.18, 10.01 and 12.45 were configured using 250 mL of deionized water and a pH buffer powder. Several tests are performed by connecting the sensor to the test circuit. The pH value of the solution is converted into a voltage value to evaluate the sensor’s performance. The pH glass electrode in a HANA HI98194 multi-parameter water quality tester is used as a standard pH meter. For HANA HI98194, the protection solution is the reference solution inside the electrode, which is immersed in the solution for a long time. Our sensors use a metal oxide tantalum pentoxide as a sensitive layer, which is stable and non-oxidizable. In contrast to glass electrodes, our sensors do not require long-term immersion in neutral solutions containing saturated KCl solutions, but only need to be immersed in the solution for a few minutes before testing to achieve an electrochemical equilibrium. When testing solutions of different pH values, deionized water should be used to clean the electrode surface of the sensor. We generally choose natural drying or air gun drying for the sensor surface moisture to avoid the cross-contamination of different solutions. Using a paper towel to wipe the surface of the sensor is not recommended, because the surface of the paper tissue can scratch and damage the pH-sensitive glass membrane, remove the gel-layer and create an electrostatic charge on the electrode. This electrostatic charge will induce instabilities on the measured signal, increasing the electrode’s response time.

In the following, a comprehensive test of the sensitivity performance of the sensor is described. The performance of pH sensors strongly depends on their response time, sensitivity, repeatability, accuracy and non-ideal effects such as hysteresis and drift.

#### 4.2.1. Response Time and Sensitivity

**Figure 6:**
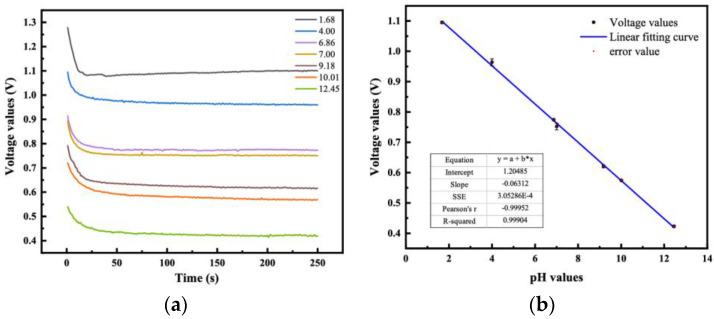
(**a**) Characteristics of response time tests in solutions at different pH values; (**b**) Linearity and sensitivity test of pH solutions from 2 to 12.

The response time is one of the most important parameters to evaluate the sensor’s performance. It refers to the time required for the instrument to respond to a sudden change in the measurement value. In this operation, the time taken for the instrument to reach 63% of the final value of the output voltage change is a fixed value. The response characteristics of the proposed sensor in different buffer solutions are systematically analyzed. The rate of change curve of the output voltage of varying pH solutions is shown in Figure 6a. After an initial rapid response, a ramp-up process is required to reach the final stability. Interestingly, the response time in an alkaline solution is slower than that in an acidic solution. In an alkaline solution, the hydrogen ion concentration is low, and the voltage drop between the signal electrode and the reference electrode is much smaller than that in the acidic solution. The time required for the output signal to reach 63% of its equilibrium state is 11.25 s. The output voltage value measured by the sensor has an excellent linear relationship with the pH value. Because hydrogen ions in a standard buffer solution with a pH value of 1.68~12.45 are always in motion, the number changes when the instrument is in the measuring state, the measurement speed is accelerated by stirring evenly, and then the test is carried out when the reading becomes stable after stirring, and the average value of the data for a consecutive 3 min is taken as the measurement value of the current pH solution, and so on. Then, the least square method was used for linear regression. This can be noted in Figure 6b for a pH value of the solution in the measurement range of 2~12 and R^2^ = 0.99904, a sensitivity value of 63.12 mV/pH is extracted, showing a Nernst response. When testing different sensor cores manufactured in the same batch, the existence of other phenomena, including an increase in the response time of the electrode, a decrease in the slope, or a zero-point shift, can either be attributed to contamination with the measuring solution or subtle differences in electrode preparation.

#### 4.2.2. Repeatability and Drift Value Measurement

**Figure 7:**
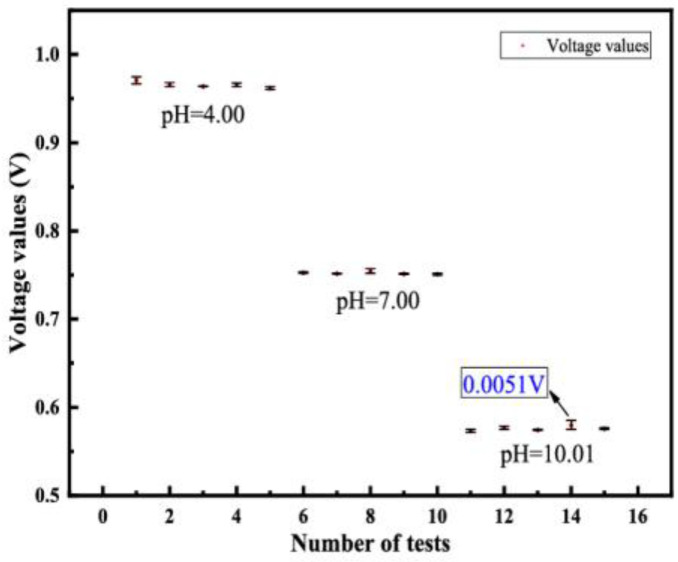
Repeatability and drift were characterized by five experiments in different solutions.

Under same working conditions, the variability of the acquired characteristic curves obtained by the continuous output change in the same direction within the entire measurement range is considered of vital significance. The repeatability of the sensor is verified by repeated testing of the sensor five times in a solution of pH values equal to 4.00, 7.00 and 10.01 at 1-min intervals. Repeatability refers to the relative standard deviation (RSD), calculated from the standard deviation and the mean of the test data, i.e., RSD is equal to the standard deviation divided by the mean. The average output curve of the repeatability test is shown in Figure 7. The maximum repeatability error is 5.29%, indicating that the pH value measurement is accurate and reliable. The drift effect refers to the slow non-random change of the output voltage of the signal electrode. The drift rate increases as the pH of the solution is increased from acidic to basic. The presence of hydroxide ions causes a higher drift in alkaline solutions than in acidic solutions. This can be observed from Figure 7, whereby the maximum drift voltage is 5.1 mV.

#### 4.2.3. Hysteresis Effect

**Figure 8:**
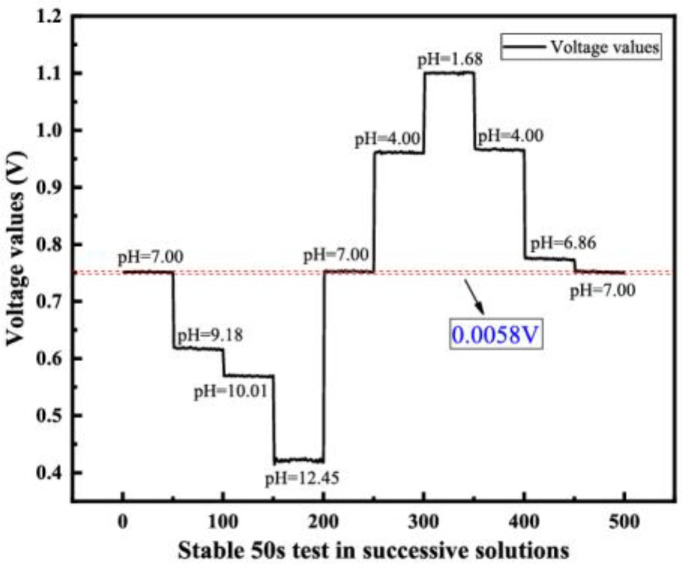
Hysteresis effect in different pH solutions.

A parameter that measures sensor characteristics is also related to hysteresis effects. The hysteresis effect refers to the degree to which the output–input characteristic curves of the sensor do not overlap during the forward and reverse strokes. A small hysteresis voltage signifies more accurate sensing measurements. The sensor’s response is measured in the following order of pH values: 7.00, 9.18, 10.01, 12.45, 7.00, 4.00, 1.68, 4.00, 6.86, 7.00. The voltage values of 50 s after the output signal stabilization in different pH solutions are selected as a ladder, and the voltage difference between the first and last pH = 7.00 solution is used as the hysteresis voltage. As Figure 8 shows, the hysteresis voltage of the sensor is less than 5.8 mV when using the straight line of precise position control in Origin software. The hysteresis width of the Ta_2_O_5_ pH sensor strongly depends on the pH measurement loop and time of measurements, the surface area and the crystalline properties of the materials.

#### 4.2.4. Performance Summary and Comparison

Table 1 compares the performance of our proposed sensing element with other sensors. It can be observed that the sensor, also made using metal oxide electrodes, has a larger measurement range and higher sensitivity than the sensor studied by Chen et al. [10]. Compared to the pH meter products, HANNA HI98194 and Seema pH838, manufactured with glass electrodes on the market, the sensitivity and response time are lower than the HANNA HI98194 products. The overall performance is better than Seema pH838 products. In this experiment, the functional film pH is connected to the processing circuit for measurement and the recursive averaging filtering algorithm is processed. The sensor has a high sensitivity and accuracy, which verifies the feasibility of the capacitive pH sensor and test circuit based on the Ta_2_O_5_ film.

## 5. Conclusions

In this work, a capacitive pH sensor is designed and thoroughly characterized. The Ta_2_O_5_ functional film is deposited through a reactive magnetron sputtering process. The surface of the prepared sensitive film is dense and uniform, with a small roughness value. When the sensor is connected to the low-power differential amplifier circuit, good linearity (R^2^ = 0.99904) and Nernst response (63.12 mV/pH) in the range of pH 2~12 is recorded. It can be concluded from the experimental results that the overall performance of the self-developed sensor is comparable to that of other sensors. The sensor has the advantages of good linearity, easy miniaturization and low preparation cost, which permitted the accurate measurement of pH changes in a water environment. In the practical application stage, it is also necessary to consider the influence of many factors, such as interfering ions (Cu^2+^, Fe^2+^ and Fe^3+^), enzyme activity effects, microbial attachment, etc. In the future, long-term stability of the proposed sensor will be tested, and it will be applied for the long-term monitoring of pH changes in the marine environment and aquaculture systems.

## Figures and Tables

**Table 1:** Comparison of different pH sensors.

pH Sensor	HANNA HI98194	Seema pH838	Chen M. et al. [10]	Self-Developed
Electrode type	Glass	Glass	Ta_2_O_5_-EIOS	Ta_2_O_5_-EIOS
Range/(pH)	0–14	0–14	1–10	2–12
Sensitivity/(mV/pH)	85.71	-	56.19	63.12
Response time/(s)	5	60	-	11.25
Drift value/(mV)	2	30	3	5.1
Hysteresis/(mV)	1	-	5	5.8

## Data Availability

The data presented in this study are available on request from the corresponding author.
